# 

**Published:** 1915-01-16

**Authors:** 


### Doctor's Wills.

The personal estate of the late Sir Henry Littlejohn, Medical Officer of Health for the City of Edinburgh, amounts to £26,624. The duty paid was £1,791.

Lieut.-Col. Charles Dalton, R.A.M.C., who died at Vieil Arcy, France, on September 18 from wounds, has left personal estate in the United Kingdom valued at £4,412 3s. 2d.

Major Edwin Bedford Steel, R.A.M.C., Assistant Director of Medical Services with the 1st Cavalry Division, who had been mentioned in dispatches for services with the Field Ambulance at Army Headquarters, and who died on November 23 last of wounds, left unsettled property of the gross value of £1,192, with net personalty £1,176.

### A COSTERS' MARATHON.

The Costers' Annual Marathon took place as usual on Boxing Day from Cambridge to Bottisham and back, a distance of about twelve miles. There were eight competitors, who had to push their coster-barrows over the twelve-mile course, which they did in spite of the rain, sleet, and snow which fell. The recent heavy rain had reduced the road to such a condition as made the task anything but a light one. A collection is usually taken on the way in aid of the funds of Addenbrooke's Hospital, and, in spite of the inclemency of the weather, the sum of £3 9s. 9d. was collected.

### Buildings Contemplated.

Gransha.—The committee of management of the Londonderry Mental Hospital have approved plans for new farm buildings at Gransha. The cost is estimated at £2,500, and tenders will be obtained for the work.

Lancaster.—The Town Council have decided to adapt a stone building built for smallpox purposes for tuberculosis and diphtheria patients. The alterations will cost £1,000.

Llandough.—The Cardiff Guardians have under consideration plans for the proposed new hospital at Llandough, which is estimated to cost £83,363. It is desired to effect certain modifications in these plans, and the building committee has been instructed by the Board to confer with the architect in the matter.

Macclesfield.—The Corporation have decided to apply to the Local Government Board for sanction to borrow £8,689 12s. 6d. for a new cubicle and administrative blocks, etc., and drainage at the isolation hospitals in Moss Lane. The plans have been prepared by the Borough Surveyor.

Waddon.—The Croydon Corporation have decided to apply for sanction to borrow £50,000 for the extension of the borough hospital at Waddon.

Wednesbury.—The architect to the Staffordshire, Wolverhampton, and Dudley Joint Tuberculosis Committee has been authorised to obtain tenders for the new dispensary buildings

### A Medical Officer Imprisoned in Germany.

Mr. Angus Macnab, the consultant for ophthalmic cases to the institutions of the Metropolitan Asylums Board, was reported to have been killed while serving at the Front with the London Scottish Territorial Regiment, but according to a later report he was not killed, but is a prisoner of war in Germany. In either case, the Board found it necessary that arrangements should be made for his duties to be carried out in his absence. The Asylums Committee communicated with Mr. Treacher Collins, the Board's ophthalmic surgeon, who, after interviewing two candidates recommended by him, appointed Mr. L. J. Pisani, F.R.C.S. Eng., to the post until March 31. Mr. Pisani is at present ophthalmic surgeon at the Metropolitan Hospital, and chief clinical assistant at the Royal London Ophthalmic Hospital. He was Llewellyn scholar and assistant demonstrator in anatomy and physiology at Charing Cross Hospital, and clinical assistant in the ophthalmic department of Charing Cross Hospital. He is a Fellow of the Royal Society of Medicine, and a member of the Ophthalmological Society. Mr. Pisani is a lieutenant-colonel (retired) in the I.M.S. During his residence in India for over twenty years, he had considerable operative experience.

### Medical Appointments.

The Metropolitan Asylums Board have made the following appointments as assistant medical officers in the Infectious Hospitals Service: Mr. C. F. Fearnside, Mr. G. Jones, Mr. F. de C. Keogh, Mr. J. M. I. A. Levins, and Mr. W. B. Tannahill.

Dr. Nicholas M. Donnelly, M.B., B.S., B.A.O. (Ireland), has been appointed senior assistant medical superintendent of the St. Pancras South Infirmary, London, and Miss M. K. Bishopp, M.R.C.S., L.R.C.P., has been appointed junior assistant medical superintendent at the same institution.

Dr. W. B. Robinson, B.A., M.D., B.Ch. Oxon., M.R.C.S. Eng., L.R.C.P. Lond., has been appointed by the Metropolitan Asylums Board medical officer at the St. George's Home for Tuberculosis for six months. The Board originally decided that the appointment should be given to an assistant medical officer at the Western Hospital, and that a local practitioner should visit when the assistant medical officer was absent on leave. The assistant medical officer having resigned, the Board were compelled to appoint Dr. Robinson, who has acted as the *locum tenens* for some time.

### Tuberculosis Treatment in Middlesex.

The Middlesex Insurance Committee have again considered the question of the remuneration to medical practitioners for treatment accorded to tuberculous patients at their own homes—the subject, as we recorded in our issue of December 19, having been referred back for further consideration at the last meeting. The Sub-Committee then suggested that the Insurance Commissioners should be informed that the payment to panel practitioners for domiciliary treatment was not equitable, and that one capitation fee should cover all medical services rendered. (The Hospital, December 19.)

At last week's meeting of the Middlesex Committee the Sanatoria Sub-Committee reported that they now recommended that the Insurance Commissioners be informed that the money allotted for tuberculosis treatment was inadequate, and further that a grant should be made to enable more adequate treatment being given to Middlesex patients.

In the subsequent discussion one member stated that it was generally recognised that the present method of payment was inequitable, and regretted that the Sub-Committee had now made no mention of this.

This was opposed, on the grounds that, in the light of experience, tuberculosis treatment would eventually have to be dealt with by the Public Health Authorities, and if a capitation fee included all ailments, compensation to doctors would have to be given on a larger scale than if the funds are kept separate as at present.

The Middlesex Council have already in hand plans for a large sanatorium, and this will afford much needed institutional treatment for patients in the country; but even with this added number of beds available, the fact is clear that the National Health Insurance Act, as at present administered, really only treats adequately a fraction of needy cases, the voluntary hospitals being taken advantage of when a case becomes outside the area of "home" treatment.

### Gifts and Bequests.

Queen Alexandra has sent a donation of £10 to the Soldiers' Dental Aid Fund.

The King has sent twenty pheasants for the sick and wounded soldiers at the Royal Southern Hospital, Liverpool.

The Queen has made a contribution to the funds of the Glasgow Royal Maternity and Women's Hospital, of which Her Majesty is the patroness.

Mr. James Edward Taylor Wainwright, of Bidston, Cheshire, and of Liverpool, surveyor and valuer, has left £100 each to the Birkenhead Borough Hospital and to the Birkenhead and Wirrall Children's Hospital.

Alderman William Radcliffe, J.P., of "Roselands," Aigburth, Liverpool, for thirty-six years a member of the committee, left £1,000 to the Royal Southern Hospital, Liverpool, and Mr. W. C. Collinson, of "Ingledene," Frodsham, Cheshire, has left £100.

The late Mrs. John Birkmyre, of Broadstone, has bequeathed £5,000 to Broadstone Jubilee Hospital, Port Glasgow. The hospital was the gift of Mr. and Mrs. John Birkmyre, and the total amount expended by them on the institution is understood to have exceeded £50,000.

The late Mr. John Henry Burley, of Leamington, Warwickshire, whose will attracted public attention from his numerous bequests, including £21,000 for his housekeeper, has left £5,000 to the Leicester Royal Infirmary for a Burley Ward, or for beds to be called Burley beds; £2,000 to the Leicester Children's Hospital; and £2,000 to the Cancer Hospital, Fulham.

The late Mr. J. M. Garrs, of North Ormesby, Middlesbrough, has left to the North Ormesby Hospital £400, North Riding Infirmary £300, Convalescent Home, Redcar, £250; to the Middlesbrough Nursing Association £100, and on the death of an annuitant one-third of the residue of his estate to North Ormesby Hospital, one-third to the North Riding Infirmary, and one-third between the Convalescent Home, Redcar, and Dr. Barnardo's Homes.

### Obituary.

Mr. Lionel G. Coplestone, son of Mr. Wm. Coplestone, local secretary of the Church of England Temperance Society, who was apprenticed in the pharmacy at Addenbrooke's Hospital, died after an operation at a hospital away from Cambridge.

### The Book Market

LIST OF NEW BOOKS RECEIVED FROM THE FOLLOWING PUBLISHERS:—

Aldine Publishing Co., Ltd., London: "The Life Story of Albert, King of the Belgians." By Percy Cook Bishop. Price 2d.

Longmans, Green and Co.: "The Invalid and Convalescent Cookery Book." By Alys Lowth. Price 1s. 6d. net.

The Scientific Press, Ltd.: "Lectures to Nurses." By Margaret Riddell. 3s. 6d. net. "Legal Engagement and Case Book for Midwives and Maternity Nurses." By Sarah Macdonald. Price 1s. net.

### Institutional and Public Reports.

Annual Report of the Health of the City of Sheffield for 1913. By Harold Scurfield, M.D., C.M., Medical Officer of Health.

The Livingstone College Year Book, 1915. Being a record of a year's work, and of former students in all parts of the world, and containing a review of recent progress in tropical medicine. Price 6d.

Report of the Royal Salop Infirmary for Year ended June 1914.

### Pamphlets and Papers.

The Campaign Against Syphilis (Based on Evidence given before the Royal Commission on Venereal Disease). By F. W. Giles, M.B. London: P. S. King and Son, Ltd. Price 6d. net.

Health in War Time: A Word in Season to the Women of Great Britain. The Women's Imperial Health Association, 7 Hanover Square, W.

RATES OF SUBSCRIPTION: Twelve months, 6/6; Six months, 4/-; Three months, 2/-, post free to any address in the United Kingdom. Abroad, Twelve months, 8/8; Six months, 5/-; Three months, 2/6, post free.—Address The Subscription Dept., "THE HOSPITAL," The Hospital Building, 28/29 Southampton Street, Strand, London, W.C.

